# Optimum PZT Patch Size for Corrosion Detection in Reinforced Concrete Using the Electromechanical Impedance Technique

**DOI:** 10.3390/s21113903

**Published:** 2021-06-05

**Authors:** Jaamac Hassan Hire, Seyedsina Hosseini, Farshad Moradi

**Affiliations:** Integrated Nanoelectronics, Department of Electrical Engineering, Aarhus University, 8200 Aarhus, Denmark; seyho@dtu.dk (S.H.); moradi@ece.au.dk (F.M.)

**Keywords:** electromechanical impedance, PZT, corrosion, reinforced concrete, structural health monitoring

## Abstract

This paper proposes the use of a 1-dimensional (1-D) electromechanical impedance model to extract proper design guidelines when selecting patch-size and frequency range for corrosion detection in reinforced concrete structures using the electromechanical impedance (EMI) technique. The theoretical results show that the sensitivity mainly lies in the peak frequencies of the impedance spectrum, while outside resonant frequencies the sensitivity levels are low, and are prone to natural variation. If the mechanical impedance ratio between the host structure and patch is too large, the peaks and thereby the sensitivity decreases. This can be counteracted by increasing the patch thickness. Tests were carried out in reinforced concrete structures, where lead zirconate titanate (PZT) patches were attached to the rebars. Patches measuring 10 × 10 mm in length and width, with thicknesses of 0.3, 0.5 and 1.5 mm, were used. The results show that only the 10 × 10 × 1.5 mm patch, was able to generate a clear peak in the 50 kHz to 400 kHz impedance spectrum. Furthermore, a reinforced concrete structure with the 1.5 mm patch attached was induced significant corrosion damages, resulting in cracking of the structure. Due to this, a leftward shift of the main peak, and creation of new peaks in the spectrum was observed.

## 1. Introduction

Reinforced concrete (RC) is the most used construction material for bridges, buildings, oil platforms and tunnels. In general, reinforced concrete is a very durable material that can withstand a large range of severe environments including marine and industrial conditions. Even though most of these structures show good, long-term performance and high durability, there are still many failures in concrete structures because of premature reinforcement corrosion leading to the need for a very costly repair or demolition of the structure [1]. Globally, corrosion in reinforced concrete (RC) is a grand challenge; it is estimated that approximately €2.3 trillion is spent annually on remediating corrosion problems, amounting to 3–4 percent of the gross world product (GWP) [2]. Corrosion in RC can also be a huge safety hazard if not properly managed and monitored. In August 2018, the one-kilometer-long and 45-m-high Morandi bridge in Italy collapsed, and 43 people were killed. Investigators have found evidence that undetected corrosion was to blame for this tragic event [3].

Corrosion is typically defined as the destructive result of a chemical reaction between a metal and its environment [4]. The corrosion of the steel reinforcement will appear in different ways, ranging from widespread uniform corrosion to very localized attacks called pitting corrosion.

For reinforcement in marine environment, a typical reaction with oxygen and water can look like [1]:

(1) $$2Fe\to \;2Fe^{2+}+4e^{-}\;\left(Anodic\;reaction\right)$$

(2) $$O_{2}+2H_{2}O+4e^{-}\to \;4OH^{-}\left(Cathodic\;reaction\right)$$

(3) $$2Fe+2H_{2}O+O_{2}\to \;2Fe\left(OH_{2}\right)\;\left(sum\;of\;the\;reactions\right)$$

Equation (3) shows that iron and hydroxide ions react together to form solid iron hydroxide, also known as rust. These complex iron oxides products can evolve according to the local environment. Depending on their level of oxidation and availability of moisture, the corrosion products will have specific volumes ranging from about two to six times that of the iron consumed. The main damage of RC structures is therefore typically not caused by loss of steel cross-section but cracking of the concrete cover due to expansive stresses exerted by the continued deposition of corrosion products, near the steel–concrete interface [5]. The cracks lead to even more deterioration since the steel is more exposed to air and the ingress of chlorides.

It is impossible to prevent corrosion of steel. Therefore, there is a need to know how long a structure can serve safely and efficiently. NACE international estimates that up to 35% of the cost of corrosion could be reduced by implementing corrosion mitigation methods like corrosion monitoring.

If corrosion is detected in an early stage, appropriate actions can be taken, to maintain or even increase the service life of structures. Repair and maintenance solutions such as the use of Fiber-reinforced polymer (FRP) [6] or impressed cathodic protection (CP) [7] can be initiated.

Today’s existing corrosion sensors, which are based on traditional non-destructive evaluation (NDE) methodologies suffer from several shortcomings for contemporary demands, such as lack of precision, extensive labor work requirement, low durability, high-power usage and high cost, as described in [8,9]. These assessment methods are usually based on electrochemical principles, such as Linear Polarization Resistance (LPR), Open Circuit Potential (OCP) and Electrical Resistance (ER) [10].

The main goal in corrosion assessment is to have quantitative results, such as the corrosion-rate, which is defined as the mass loss of the steel per unit area per unit time (typically mm/year). In the LPR method only an indicative estimation of the corrosion-rate, can be calculated, while the OCP only gives qualitative results based on the probability of the presence of corrosion.

Due to the several drawbacks of the commercially available electrochemical sensors, there is currently great technical and scientific interest in research and development into more accurate, permanent, embeddable, self-sufficient and wireless corrosion sensors. The scientific literature describes several novel sensing principles that can be embedded inside the concrete. The alternative sensing methods are based on capacitance [11], magnetic [12] and optical properties [13]. Unfortunately, many of these methods are still in the early stages of research.

Novel Structural Health Monitoring (SHM) systems based on piezoelectrical principles have recently been studied extensively by many research groups, as in [14,15,16] and [17]. It can detect and quantify incipient hazardous damages due to corrosion or cracks. It is an emerging research area with multiple applications in a variety of critical infrastructures and vehicular structures, such as bridges, oil platforms, highways, ships and aircrafts, and is on the edge of industrial application. Piezo-based SHM can be performed using several methodologies: (a) wave propagation, (b) frequency response transfer function and (c) electromechanical impedance (EMI) [18]. The principles can be used in conjunction with Wireless Sensor Node (WSN) and energy harvesting configurations for a sustainable real-time monitoring.

In this paper, only the EMI method is given attention. The EMI technique utilizes piezoelectric transducers, where in most cases PZT patches are used. PZTs can be characterized as small, highly sensitive, inexpensive and low power smart materials. The EMI method for corrosion monitoring in reinforced concrete has been investigated by a few research groups. Talakokula et al. [19] have shown that it is possible to evaluate the corrosion process in reinforced concrete, by embedding PZT patches to the rebar. Their experimental results showed that the method is not only effective in detecting incipient corrosion, but also quantifying the amount. Weijie li. et al. [20] have also shown that the EMI method can be effectively used to determine the corrosion amount. Furthermore Ahmadi et al. [21] have recently developed a model to obtain the corrosion rate in reinforced concrete from the impedance values.

The study presented here will be on the investigation of the optimum sizing of the piezo-electrical patch for optimum corrosion detection in reinforced concrete, which is done theoretically and experimentally. The results indicate that high levels of sensitivity can be obtained if resonant modes of the structure are excited in the impedance spectrum. But large differences between the mechanical impedance of the host structure and PZT patch, as in reinforced concrete, will dampen the peak areas. To counteract this, the patch thickness can be increased.

This paper is organized as follows. In Section 2, the basic principles of the EMI are introduced, furthermore a theoretical analysis and review of the correct patch sizing and frequency are made. In Section 3, experimental impedance measurements of both steel in free air and in RC are presented. In Section 4, the theoretical, experimental results and possible real-life use of EMI in RC are discussed. Finally, the paper ends with a conclusion in Section 5.

## 2. Electromechanical Impedance Theory

### 2.1. Background

**Figure 1:**
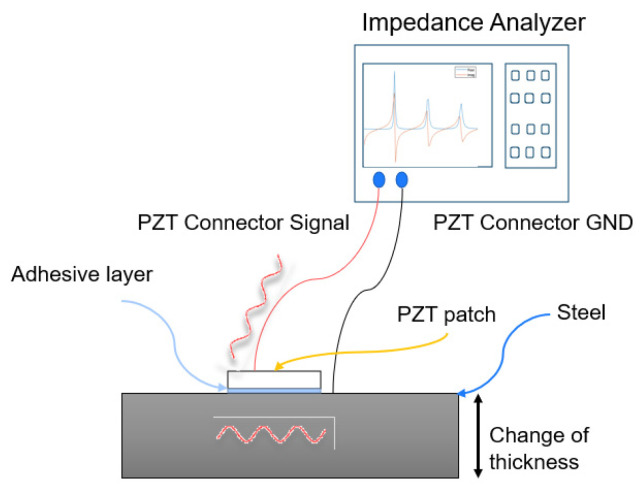
Electromechanical impedance principle.

The electromechanical interaction between the PZT patch and the host structure is the main principle of damage detection in the EMI method. An illustration of the test setup is shown in Figure 1. The bottom electrode of the PZT patch is attached to the host structure (steel) with a thin layer of conductive epoxy, so they are strongly coupled. Wires from the steel (GND) and the top electrode of the PZT patch (Signal) are led to an impedance analyzer.

The measurements by the impedance analyzer, involves exciting a sinusoidal voltage across the square bonded PZT patch. The deformations are produced both in the patch and in the host structure. The patch will induce elastic waves into the beam structure, which will reflect at the beam boundaries and set it into oscillation [18]. The imposed mechanical vibrations are then transferred back to the PZT patch and is then reflected in the electrical impedance spectrum as a peaks and valleys signature.

Liang et al. [22] were the first to propose a 1-dimensional (1-D) analytical model to analyze the electromechanical interaction between a PZT patch and a host structure that predicts the electrical impedance. The equation for the electrical admittance that is measured at the terminal of the patch can be written as:

(4) $$\bar{Y}=\omega j\frac{wl}{h}\left[\left(\bar{\varepsilon_{33}^{T}}-d_{31}^{2}\bar{Y^{E}\;}\right)+\left(\frac{Z_{T}}{Z_{S}+Z_{T}}\right)d_{31}^{2}\bar{Y^{E}\;}\left(\frac{\tan\left(\kappa l\right)}{kl}\right)\right]$$

where the impedance is *Z* = 1/*Y*.

*w*, *h* and *l* are width, height and length (the dimensions of the PZT patch), $\bar{\varepsilon_{33}^{T}}$ is the complex electric permittivity, *d*_31_ is the piezoelectric strain coefficient, $\bar{Y^{E}\;}$ is the complex young modulus, and *κ* is the 1-D wave number related to the angular frequency of the excitation signal *ω*. The full description of the parameters can be found in [23].

The equation also shows that the electrical admittance/impedance is dependent on the mechanical impedance of the patch *Z_T_* and the host structure *Z_S_*, respectively. The mechanical structural impedance, *Z_S_*, can be modelled as a frequency dependent 1-D spring-mass-damper system [24], where:

(5) $$Z_{S}\left(\omega \right)=c+m\omega j-\frac{kj}{\omega }$$

where *‘c’* is the damping constant, *‘m’* the mass and *‘k’* the stiffness of the host structure. Damage to the structure will inflict changes in these structural properties, i.e., the stiffness and mass. Since *Z_T_* is typically much smaller than *Z_S_*, and the other parameters are fixed, any change in the electrical impedance will be due to *Z_S_*.

Another EMI model based on a modified Mason’s model has been suggested by Baptista et al.:

(6) $$Z_{E}=\frac{1}{j\omega C_{0}}||jZ_{T}{\left(\frac{s_{11}}{d_{31}\cdot L}\right)}^{2}\cdot \left[\frac{1}{2}\cdot \tan\frac{\left(k\cdot L\right)}{2}-\frac{1}{\sin\left(k\cdot L\right)}+\frac{Z_{s}}{j2Z_{T}}\right]$$

where *Z_E_* is the electrical impedance, *Z_S_* is the mechanical impedance of the host structure and *Z_T_* is the mechanical impedance of the PZT patch. The full description of the parameters can be found in [25].

However, the Liang and Baptista et al. models do not include modelling of the structural substrate, which makes comparisons with experimental results directly difficult. The work of Giurgiutiu et al. [26] is one of the few that extensively treats the modeling of the electromechanical impedance technique in details. In their paper, Giurgiutiu et al. drive an analytical model based on the structural vibration and the theory of piezoelectricity to predict the EMI impedance response, where the modelling of the substrate is included. They have obtained good theoretical results, which corresponds well with their experimental measurements.

The analytical model considers a 1-D structure. The electrical impedance as measured at the terminals of the patch is given by,

(7) $$Z_{E}=\frac{1}{j\omega C^{E}}{\left[1-\kappa_{31}^{2}\left(1-\frac{1}{\varphi \cot\left(\varphi \right)+r}\right)\right]}^{-1}$$

*ω* is the angular frequency and *C^E^* is the capacitance with electrical loss factor defined as:

(8) $$C^{E}=C\cdot \left(1-j\delta \right)$$

where *δ* is electrical loss factor in %. *C* being the capacitive factor, defined as:

(9) $$C=\varepsilon_{33}\cdot b_{p}\cdot \frac{l_{p}}{t_{p}},$$

where *ε*_33_ is dielectric constant, *b_p_* is the patch width, *t_p_* is the thickness and *l_p_* is the length.

$\kappa_{31}$ is complex coupling factor defined as:

(10) $$\kappa_{31}^{2}=\frac{d_{31}^{2}}{s_{11}\varepsilon_{33}}$$

*d*_31_ is the in-plane induced-strain. $s_{11}^{E}$ is the compliance (with mechanical loss) defined as:

(11) $$s_{11}^{E}=s_{11}\cdot \left(1-j\eta \right)$$

*s*_11_ is compliance without loss factor, *η* is the mechanical loss factor given in %. φcot(φ) is the resonant term and defined as $\varphi =\frac{1}{2}\cdot \omega \cdot \frac{l_{p}}{c_{-}}$. Here it is apparent that only the length, *l_p_*, of the patch size dimensions, will have an impact on the resonant term. *c*___ is the speed of sound in the material, defined as:

(12) $$c\_=\sqrt{\frac{\left(\frac{1}{\rho }\right)}{s_{11}^{E}}}$$

where *ρ* is the density of the PZT. *r* is the structural mechanical stiffness ratio and defined as:

(13) $$r\left(\omega \right)=\frac{k_{S}\left(\omega \right)}{k_{T}}$$

$k_{S}\left(\omega \right)$ is the frequency-dependent structure dynamic stiffness. It is a relationship between force ($F_{pzt}\left(\omega \right)$) and velocity ($u_{pzt}\left(\omega \right))$ (For full Equation see [26]).

(14) $$k_{S}\left(\omega \right)=\frac{F_{pzt}\left(\omega \right)}{u_{pzt}\left(\omega \right)}$$

where *k_T_* is the transducer static stiffness and is defined as:

(15) $$k_{T}=\frac{A_{a}}{s_{11}^{E}l_{a}}$$

$A_{a}=b_{p}\cdot t_{p}$ is the cross-sectional area of the patch.

All the mentioned models are based on 1-D assumptions, meaning that only deformations along the length of the structure is considered. Therefore, the models cannot give a full description of the interaction between the patch and the host structure, which presents very complex vibration characteristics. However, they can help obtaining a partial picture of the complex interaction.

### 2.2. Optimal Frequency and Patch Sizing Selection

The sensitivity of detecting damage through EMI is closely related to the selected frequency band. For the method to be sensitive to small cracks and damages, it is necessary that the wavelength of the excitation signal to be smaller than the characteristic wavelength of the damage to be detected. The excitation range should include a high enough number of resonance peaks, which implies that there is a great dynamic interaction between the patch and host structure. Hence it is suggested that operation frequencies from 30 kHz to 400 kHz is used to detect incipient-type damages. Due to this high frequency range, the principle is also less dependent on the boundary conditions, which makes it possible to have good repeatability between structures. Noise factors, such as environmental and mechanical vibrations do typically not extend into the range of tens of kHz, and therefore, these disturbances will have little to no effect on the sensitivity of the sensor [27]. The dimensions of the patch are highly related to what kind of resonance modes can be excited in the host structure. The suggestions are sizes (length and width) ranging from 5 to 20 mm and thicknesses of 0.1 to 0.3 mm are best suited for most structures [28] such as steel and reinforced concrete. These frequency ranges and sizes are typically determined by trial and error methods, while little analytical work is done regarding correct patch sizing and frequency selection for optimum sensing. To our best of knowledge, only work by Baptista et al., have used theoretically derived methodologies in [25,29,30] to determine parameters for optimum sensing. In their methodology, they do not include the resonant frequencies of neither the host structure nor the PZT patch. They have obtained good results for frequencies below 125 kHz but are lacking results for higher frequencies.

The study presented in this paper uses similar methodologies but incorporates the higher resonant frequencies of the EMI, where it is more useful, using Giurgiutiu et al.’s model.

Using the self-actuation and sensing capabilities of the PZT-patch, the damage detection is performed by analyzing the variations in magnitude and shifts in frequency of the electrical impedance/admittance measured at the terminals of the patch using an impedance analyzer. To quantify the damage level, impedance measurements of the structure in healthy (pristine) state called baseline, is obtained, and then compared with the impedance measurements in damage state. Damage metrics such as root mean square deviation (RMSD) and correlation coefficient deviation mean (CCDM) or just detecting changes in the resonance frequency are often used.

The mechanical impedance/stiffness of the host structure *Z_S_* and *K_S_* is typically many times larger than the mechanical impedance/stiffness of the patch *Z_T_* and *K_T_*. Variations in the electrical impedance *Z_E_*, is therefore an indication of damage occurrence in the host structure.

The goal is to find the optimum size of the patch that will give the maximum sensitivity of the sensor. This means that a small change of *Z_S_* (*K_S_*), should result in a detectable change in *Z_E_*_._ As described by Baptista et al. [25], it is useful to examine changes in *Z_E_* as a function of the ratio between the mechanical impedance of the host structure to the patch as *Z_S_*/*Z_T_* or mechanical stiffness *K_S_*/*K_T_*_._ In this paper, it is considered that those two entities are the same (they are both a relationship between force and velocity); for simplicity they are from now on, defined as the loading.

For example, by mathematically looking at Giurgiutiu et al.’s model in Equation (7), specifically, the stiffness ratio at Equation (16), and for simplicity assuming that *k_S_* is not frequency dependent, it is possible to deduce the patch sensitivity information:

(16) $$r=\frac{k_{S}}{k_{T}}$$

If *k_S_* is much larger than *k_T_*, *r* value becomes very large. If it is large enough, the resonant term, φcot(φ), will not have any more influence on *Z_E_*. The intrinsic parameters, especially the capacitive component of the patch, will become more dominant, therefore no changes due to damages in the host structure can be detected.

**Figure 2:**
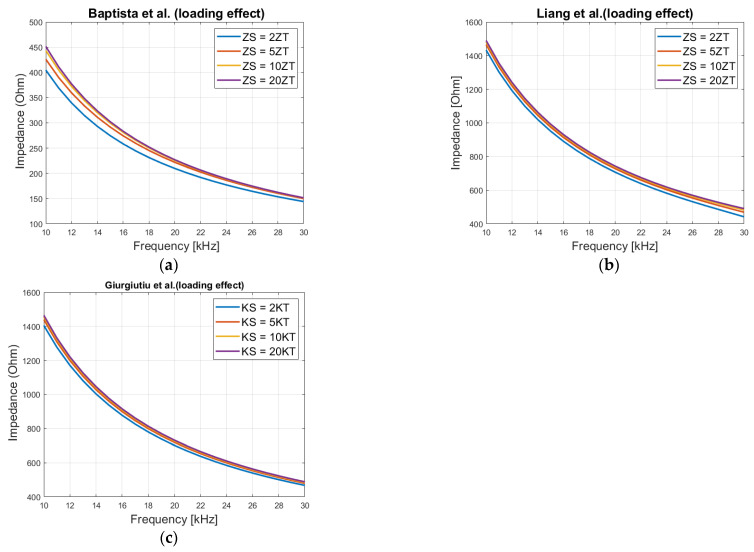
Loading effect on the electrical impedance, (**a**) Baptista et al. model, (**b**) Liang et al. model and (**c**) Giurgiutiu et al. model.

For analyzing this effect graphically (theoretical), first at areas where no resonant peaks exist, the loading *K_S_* (*Z_S_*) is increased from 2*K_T_* (*Z_T_*) to 20*K_T_* (*Z_T_*). To calculate the modulus of the impedance, a frequency range of 10 kHz to 30 kHz, and a patch size of 20 mm × 20 mm × 0.5 mm is considered. The same method is performed for Liang and Baptista et al.’s models and plotted as shown in Figure 2. The patch parameters can be found in Table 1 for Giurgiutiu et al.’s model, for Liang and Baptista et al. models in [23] and [25] respectively.

It is seen that the impedance (modulus) value increases with increased loading. At higher loadings, the changes that can be observed become smaller. This counts for all three models meaning that the electrical impedance can vary between two values, minimum and maximum, depending on the size of the host structure. The minimum impedance is when there is no host structure, so *K_S_* (*Z_S_*) is zero. The maximum impedance is achieved when *K_S_* (*Z_S_*) is much larger than *K_T_* (*Z_T_*), as for large structures.

**Figure 3:**
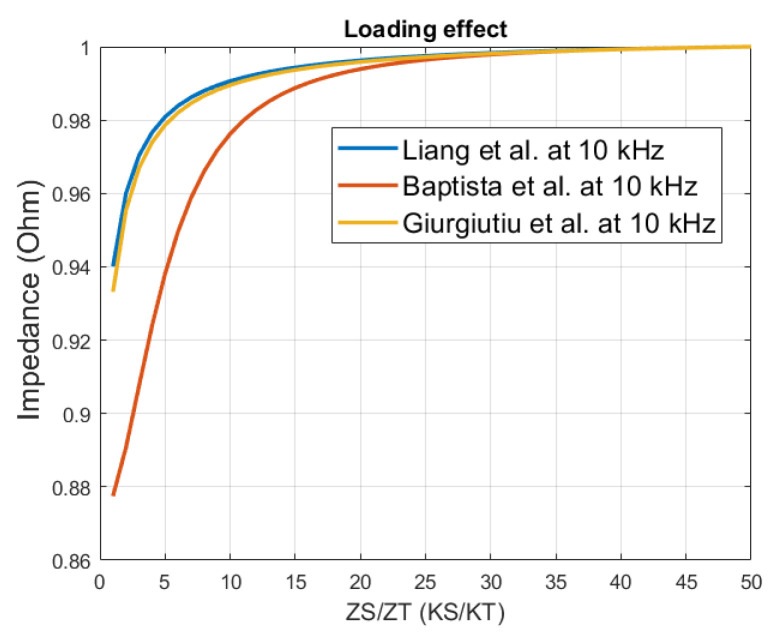
Normalized impedance values as function of the loading *ZS*/*ZT* (*KS*/*KT*) with all three models at 10 kHz.

In Figure 3, a better visual analysis of this effect is obtained by setting a fixed frequency and computing the electrical impedance as a function of the loading. The impedance value is normalized for all models.

The graph in Figure 3 shows the impedance values as a function of the loading at a fixed 10 kHz frequency for the different models. When the loading increases, the impedance increases towards its upper limit. Hence, there is no more variations in the electrical impedance, with respect to the variations in the ratio *Z_S_*/*Z_T_* (*K_S_*/*K_T_*), this behavior accounts for all three models. This means that damage detection can be more difficult in large structures if only the ‘pure’ impedance is used, which are areas that do not contain peaks.

**Figure 4:**
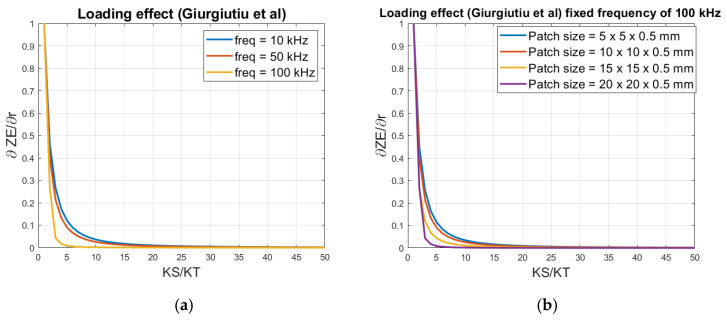
Normalized derived of the impedance as function of the loading regarding (**a**) frequency impact, and (**b**) size impact.

For comparing the loading effects at different fixed frequencies, only using Giurgiutiu et al.’s model, again outside resonant areas, the derivative of the electrical impedance (Equation (7)) with respect to the ratio *K_S_*/*K_T_* (r) is taken (Equation (17)). Then this is normalized between 0 and 1, which corresponds to the minimum and maximum impedance, respectively. The analysis is done for 10 kHz, 50 kHz and 100 kHz, as seen in Figure 4a.

(17) $$\frac{\delta Z_{E}}{\delta r}=\frac{j\cdot k_{31}}{\omega C{\left(r+\varphi \cot\left(\varphi \right)\right)}^{2}\cdot \left(k_{31}\cdot \left(\frac{1}{r+\varphi \cot\left(\varphi \right)}-1\right)+1\right)}$$

There is no noticeable difference in the slope between 10 kHz and 50 kHz, but for 100 kHz it has a slightly lower slope at the loading region between 3 and 20. This indicates, that the sensitivity decreases with higher frequencies, when only the ‘pure’ impedance spectrum is used.

Further investigations on the impact of the size of the patch, shown in Figure 4b, as a function of the loading, with a fixed frequency of 100 kHz, with following sizes of 5 mm × 5 mm × 0.5 mm, 10 mm × 10 mm × 0.5 mm, 15 mm × 15 mm × 0.5 mm and 20 mm × 20 mm × 0.5 mm. For all patch sizes, the optimum sensitivity is achieved with loading ratios below 3. The plot also shows that a smaller patch size will give a relatively higher detection range. A 5 mm × 5 mm × 0.5 mm patch can detect variations up to 15 times its own loading, while for 20 mm × 20 mm × 0.5 mm, it varies only up to 3 times its loading. Since the used Giurgiutiu et al. model is 1-D, only the length has an impact on the slope with respect to the loading variations.

**Figure 5:**
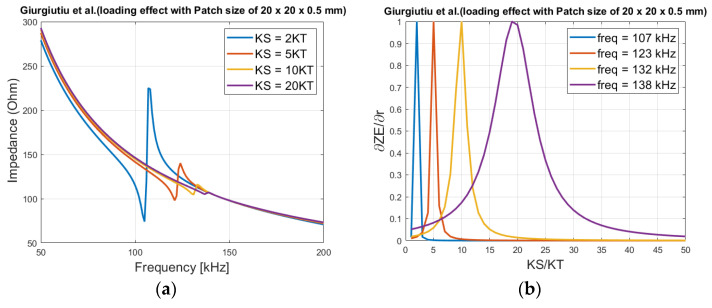
(**a**) Resonant areas of the impedance with patch size of 20 mm × 20 mm × 0.5 mm, (**b**) normalized derived of the real part of impedance as a function of the loading *KS*/*KT*.

If the analysis is done at regions where resonant frequencies exist, using a patch size of 20 mm × 20 mm × 0.5 mm, and the loading increases as shown in Figure 5a, higher loadings cause a rightward shift, and the amplitude decreases. This effect can also be observed in Liang’s model (see Equation (4)) if the stiffness parameter increases.

Plotting in the peak frequencies shown in Figure 5b at 107 kHz (*KS* = 2 *KT*), 123 kHz (KS = 5 *KT*), 132 kHz (*KS* = 10 *KT*) and 138 kHz (*KS* = 20 *KT*), for a better graphical analysis the normalized derivate of the impedance with respect to the ratio *KS*/*KT* is calculated (using Equation (17)). It shows (Figure 5b) that the impedance variation at 107 kHz has a very narrow width, and only exist for loading between 1 to 4. At the higher resonant frequencies, which is generated by larger loadings, the width broadens, meaning a higher variational range in the impedance values can be detected in the frequency. This indicates that even large damage levels can be quantified by tracking changes in the peak-frequency/frequencies instead of just using the ‘pure’ impedance as in Figure 3, where impedance value saturates with respect to *KS*/*KT* (loading).

The sensitivity is highest at 107 kHz, since the magnitude is much higher than at 138 kHz, but only for a ratio variation between 1 to 4 *KS*/*KT*.

### 2.3. Frequency Sensitivity by Thickness Variation

To investigate sensitive frequency areas, the same exercise as Baptista et al. [30] is performed. However, here, we use Giurgiutiu et al.’s model since this model includes a resonant term. A 5% variation (∆) in the mechanical stiffness ratio as (1 + ∆)r, see Equation (7), is assumed. Due to a hypothetical damage on the host structure, the percentage variation *η* of the electrical impedance before and after damage is calculated as:

(18) $$\eta =100\frac{\left[Re\left(Z_{D}\right)-Re\left(Z_{h}\right)\right]}{Re\left(Z_{h}\right)}$$

*Re*(*Z_D_*) is the real part of electrical impedance value simulated with 5 % damage and *Re*(*Z_h_*) is the healthy unaffected one. In the experiments, a PZT-patch with a length and width of 10 mm is used, therefore the same size is applied in this analysis, only the thickness of the patch is varied. An operational window from 50 to 400 kHz is chosen.

**Figure 6:**
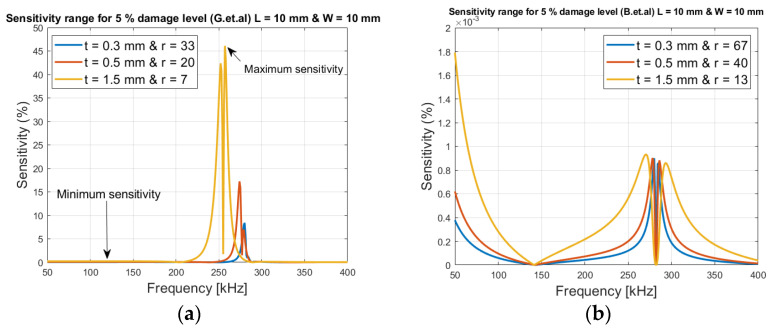
Sensitivity levels with respect to thickness variations (**a**) Giurgiutiu’s et al.’s model (**b**) Baptista et al. model.

Figure 6a shows that it is possible to obtain the best sensitivity between 210 kHz to 280 kHz, which are resonance points; outside these points the sensitivity is very low.

From Equation (15), it is observed that the mechanical impedance (stiffness) of the patch can increase if the width or thickness of the patch increases. Accordingly, the length decreases the mechanical impedance/stiffness, but it is a 1-D model and the patch is assumed to be square (width and length is the same).

From Figure 6a, where *r* is the loading ratio between *K_S_*/*K_T_*, it is shown that the sensitivity increases with increased patch thickness (t). This is due to the fact that *r* (loading) is decreased from 33 to 7, and thereby the magnitude of the resonant is increased.

According to Baptista et al. the mechanical impedance of the patch can be calculated as follows [30],

(19) $$Z_{T}=\sqrt{\frac{\rho_{T}}{s_{11}}}A_{T,}$$

where *A_T_* = *L* · *t* is the cross-sectional area of the patch, where *L* is the patch length and *t* is thickness. *ρ_T_* is the mass density of the PZT and *s*_11_ is complex compliance.

The mechanical impedance of the host structure *Z_S_* (frequency dependence is neglected), is defined as

(20) $$Z_{S}=\sqrt{\frac{\rho_{S}}{s_{s}}}A_{S,}$$

where *A_S_* = *L* · *t*, *L* is the structure length and *t* is the thickness. *ρ_S_* and *s_s_* are the mass density and compliance of the material, respectively.

As shown in Figure 6b, from Baptista et al.’s model, it is seen that the sensitivity also increases over a large frequency spectrum, when the thickness is increased, since the loading ratio *r* is decreased. Though the sensitivity levels are at much lower percentages (below 0.0018%), this is due to the fact that Baptista et al.’s model does not take the resonance frequencies into account, where high sensitivity levels can be obtained.

## 3. Experimental Results

### 3.1. Loading Effect Investigation on Small Steel Beams

**Figure 7:**
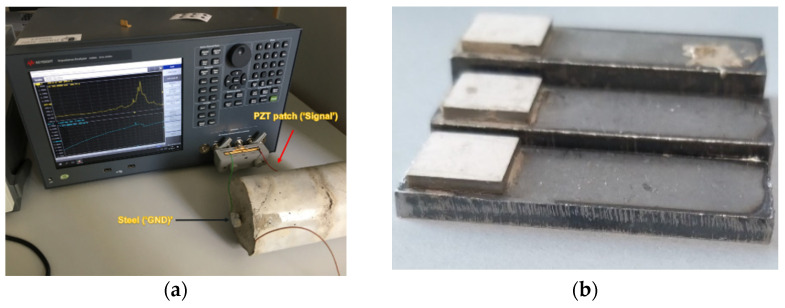
(**a**) Impedance measurement setup for reinforced concrete sample using Keysight E4990A impedance analyzer and (**b**) steel beam with different thickness.

Figure 7a shows the test setup for all the performed impedance measurements, in this case shown with a reinforced concrete sample. Keysight E4990A impedance analyzer is used, see Figure 1 and Section 2.1 for a more detailed test setup explanation.

First investigations were performed on three small steel beams, shown in Figure 7b, with length and width of 40 mm and 13 mm, respectively, to observe the changes in the impedance spectrum. The thickness of the steel beam is 3 mm, 5 mm and 8 mm. A patch size of 10 mm × 10 mm × 1.5 mm, and the real part of the impedance is used for the measurements. All the PZT patches used in this paper is from American Piezo (APC) [31] of the type 840.

**Figure 8:**
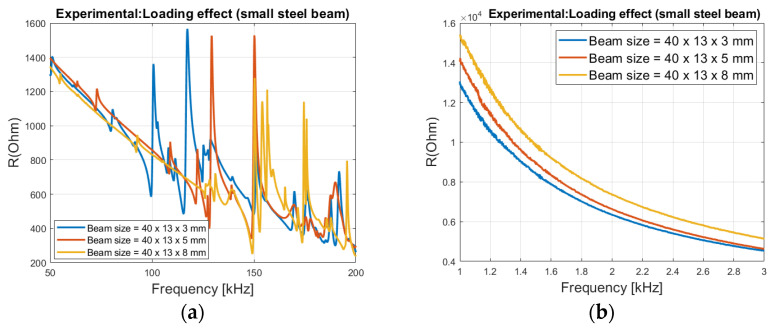
Impact of host structure size on the electrical impedance, (**a**) resonant areas and (**b**) outside resonant areas.

Figure 8a shows the resonant frequencies is shifted to the right, with higher steel beam thickness (higher loading, see Equation (20)). This agrees well with the theoretical analysis in Figure 5a. If only the ‘pure’ impedance is considered, only systematic changes at very low frequencies (1 to 3 kHz) can be observed. In Figure 8b it is shown that if the thickness of the steel beam is increased, the electrical impedance will increase. It can also be seen that the change in the impedance becomes smaller at higher frequencies. This agrees well with the theoretical analysis in Figure 2 and Figure 4a.

### 3.2. Reinforcement Steel Rod and Damage Detection in Free Air

In this test-setup, patch thicknesses were investigated for optimum detection of corrosion in reinforced concrete, where steel rods were used.

**Figure 9:**
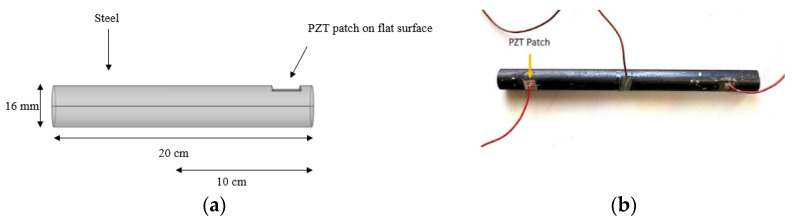
(**a**) Diagram of steel dimensions and (**b**) actual steel used in the experiments.

First, the impedance measurements of the reinforcement steel rods in free air were taken. Two steel rods of 200 mm and 100 mm long were used, both with the diameter of 16 mm as shown in Figure 9. Part of the steel rod was cut with a milling machine in a small groove (flat surface) for the placement of the PZT patch. The maximum length (L) and width (W), which could be grooved was 11 × 11 mm with a 1 mm depth. The maximum patch length and width, that could be placed on the grooved steel surface safely was 10 × 10 mm. Therefore, only the thickness was varied, with 0.3, 0.5 and 1.5 mm. The patch is attached to the steel with a thin layer of conductive epoxy.

**Figure 10:**
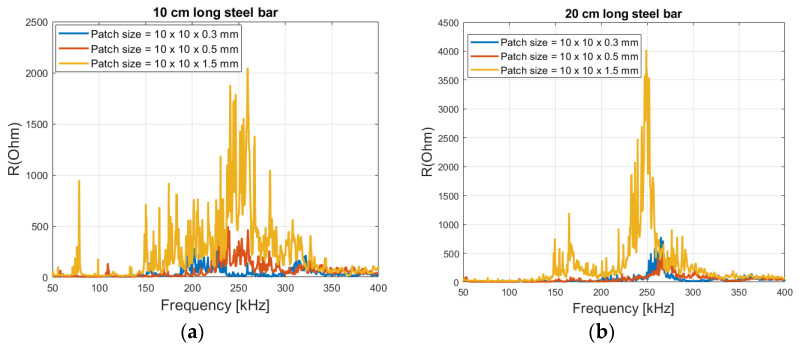
(**a**) The real part of impedance over frequency ranges from 50 to 400 kHz with 10 cm long (**b**) and 20 cm long steel beam in free air.

Figure 10 shows measurements of the real part of the impedance over a frequency from 50 kHz to 400 kHz, great dynamic activities in the region are seen for both the steel lengths and the patch thicknesses. Regarding magnitude, the difference between the patch size thickness of 0.3 and 0.5 mm is on similar scale, but an increase to 1.5 mm shows significantly higher magnitudes for both steel lengths. It should be noted that an increase in patch thickness also has the effect of decreasing the capacitance of the patch (see Equation (9)), which means higher impedance values.

**Figure 11:**
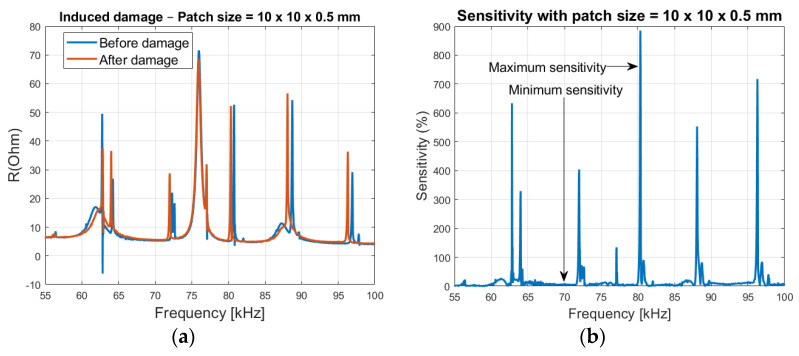
(**a**) Before and after damage on the steel rod, real part of the impedance signature (steel size = 20 × 16 mm and patch size = 10 × 10 × 0.5 mm) and (**b**) Sensitivity plot as a function of frequency.

Due to the high dynamic activities in the region, which normally is a desired feature [27], but because of the closely spaced peaks, it can cause confusion in identification of the changes in the spectrum. Therefore, a smaller window with less ‘noise’ is recommended, such as 55 to 100 kHz as shown in Figure 11.

By inducing 3–5% damage, in the 20 cm long steel rod (damage induced 16 cm from the patch location), it is seen in Figure 11, how the impedance signature results mostly in a left shift of the peak frequencies, because of stiffness loss. It can also be observed that some of the peaks are less sensitive to the induced damage-like the peak at 75.9 kHz.

By calculating the percentage variation of the electrical impedance using Equation (18), it is seen that very high sensitivity levels can be obtained in the spectrum, though only existing at resonant areas as shown in Figure 11b. This corresponds with the theoretical analysis shown in Figure 6.

### 3.3. Patch Sizing in Reinforced Concrete

**Figure 12:**
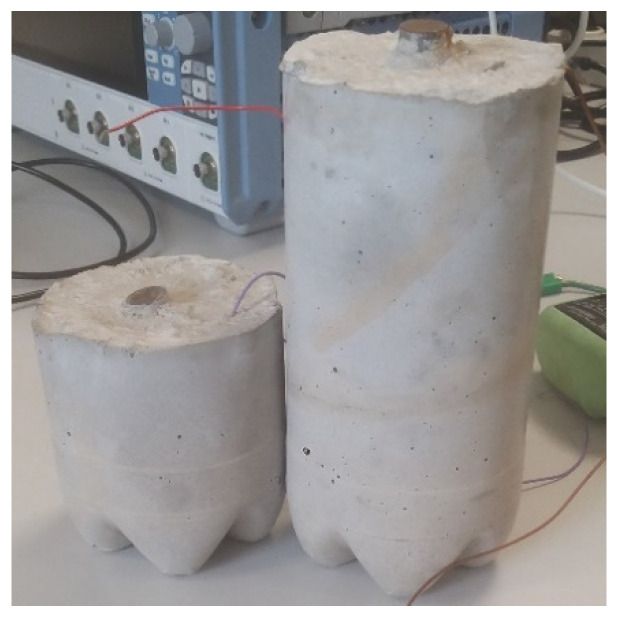
Reinforced concrete structures with 10 and 20 cm long steel with a concrete cover thickness of 40 mm.

The steel beams with the attached PZT patches were now embedded inside concrete with a concrete cover thickness of 40 mm, see Figure 12. The patches were not protected with any coating since it was expected that the concrete itself will provide the needed protection. To avoid the impact of the curing process, the impedance measurements for the baseline signature used for comparison were taken at least 1 month after casting for all patch sizes embedded inside the reinforced concrete structures.

**Figure 13:**
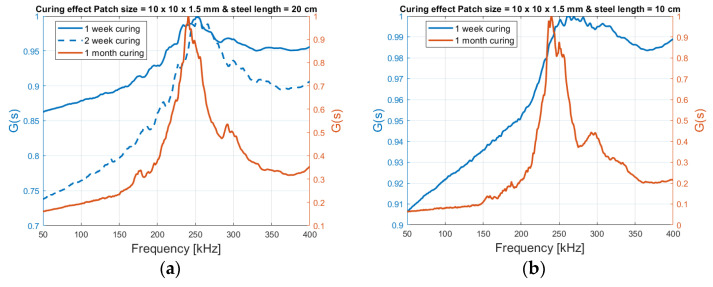
Curing effect on the admittance signature. (**a**) 20 cm long steel beam embedded in concrete with Patch size = 10 × 10 × 1.5 mm; (**b**) 10 cm long steel beam embedded in concrete with Patch size = 10 × 10 × 1.5 mm.

Figure 13 shows how the signature (admittance), with a patch size of 10 mm × 10 mm × 1.5 mm, gets more peak-shaped from week 1 after casting to 1 month for both steel lengths. The admittance values are used and normalized, to highlight the shape behavior.

Recent research has shown that this behavior can be used to track the curing process of concrete [32], where the compressive strengthening and stiffness of the concrete develops.

**Figure 14:**
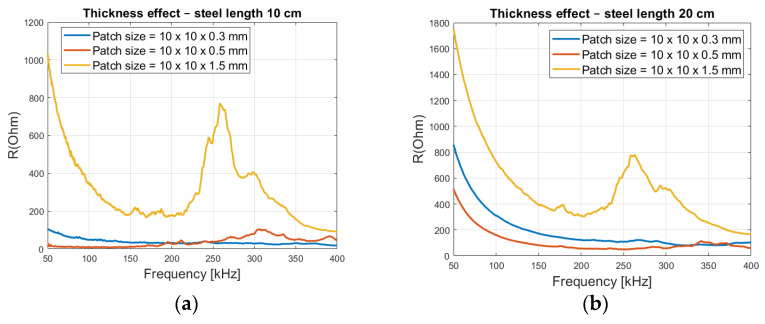
Real part of the impedance with varying patch thickness (**a**) 10 cm and (**b**) 20 cm steel rod.

From Figure 14, it is seen that a patch thickness of 0.3 mm has a flat spectrum in both steel lengths, without any peaks. 0.5 mm has a slight peak at around 320 kHz for 10 cm long steel (Figure 14a) and around 350 kHz for 20 cm long steel (Figure 14b). An increase in thickness to 1.5 mm shows a clear and strong peak at around 260 kHz for both steel lengths. This is because the loading ratio *r* (*K_S_*/*K_T_*), has been decreased allowing peaks to exist, as deduced in Figure 5a.

**Figure 15:**
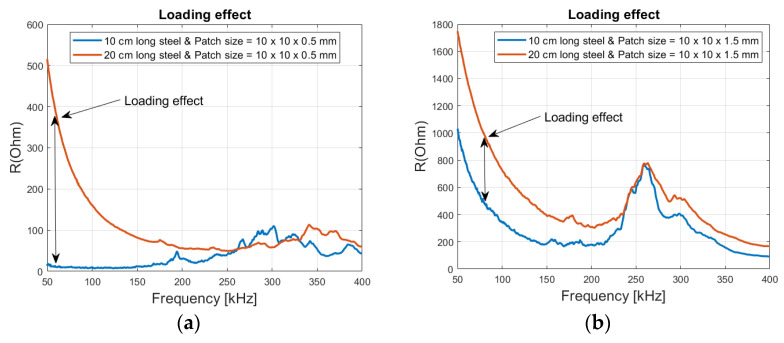
Loading effect from 10 and 20 cm long steel rod embedded inside concrete (**a**) Patch size of 10 × 10 × 0.5 mm and (**b**) Patch size of 10 × 10 × 1.5 mm.

Figure 15 shows that the loading effect is more visible in reinforced concrete, with the 0.5 mm and 1.5 mm patch thicknesses. The impedance values at lower frequencies are much higher in the 20 cm long steel beam than in the 10 cm one, due to the higher volume of concrete.

### 3.4. Accelerated Corrosion of the Reinforced Concrete

**Figure 16:**
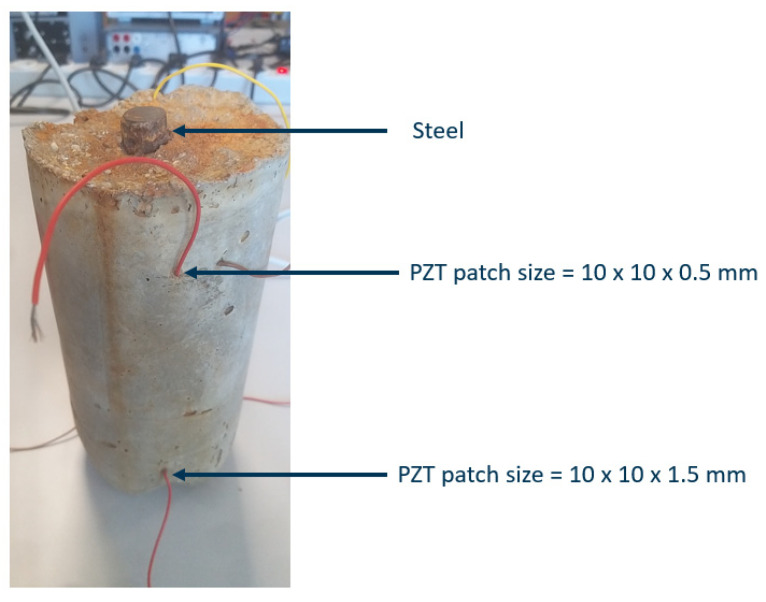
Corroded (20 cm long steel) reinforced concrete.

To investigate the sensitivity of the chosen patch sizes of 10 × 10 × 0.5 mm, placed at the top, and 10 × 10 × 1.5 mm patch placed at the bottom of the concrete as shown in Figure 16. 0.3 mm is disregarded because of its flat impedance spectrum. Only the 20 cm long steel rod embedded in concrete is corroded. An accelerated corrosion process by using the impressed current technique [19] was performed until visible amounts of corrosion resulting in cracks were obtained. As seen in Figure 16, the corrosion can be seen on the exposed part of the steel, where up to 5 mm of metal loss was measured.

**Figure 17:**
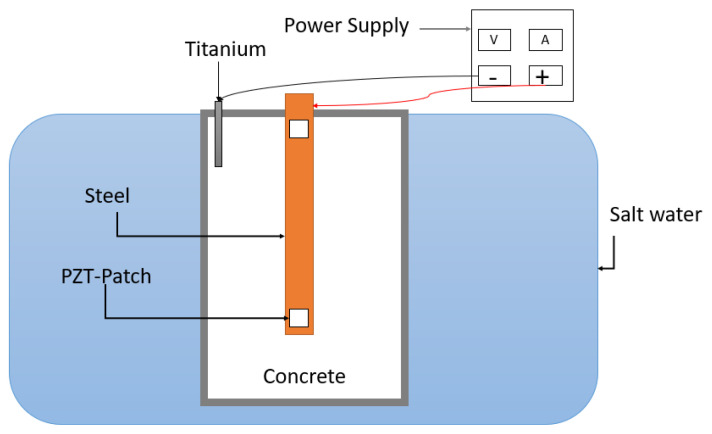
Impressed current technique for acceleration of corrosion damage in RC structure.

Figure 17 shows the test setup for the impressed current acceleration technique. The concrete is immersed in salt water. A titanium electrode working as the cathode (attached to the negative power supply) is embedded into the concrete, the steel itself works as the anode (attached to the positive power supply). By doing this, it was observed, that the corrosion acceleration process went much faster, than if the titanium cathode was placed outside the concrete. Subsequently the RC sample was dried for 5 days, before measurements were made.

**Figure 18:**
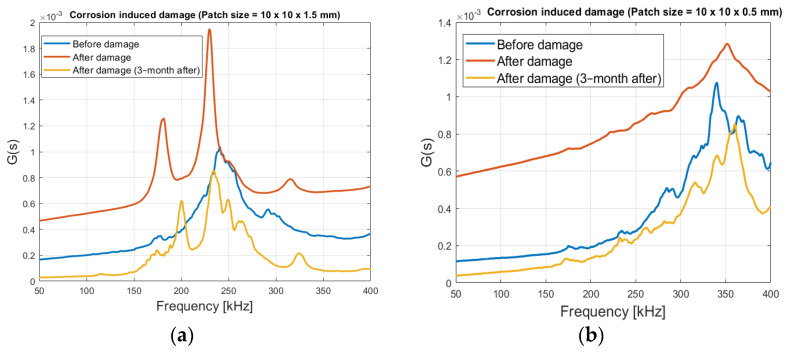
Before damage and damage signature in RC, steel size = 200 mm × 16 mm. (**a**) Patch size = 10 × 10 × 1.5 mm, and (**b**) Patch Size = 10 × 10 × 0.5 mm.

From Figure 18a, with the 1.5 mm thick patch, it is clearly seen that the (admittance) signature changes after the corrosion process. The main peak is shifted leftward from 240 kHz to 229.6 kHz and increases in magnitude, while two new peaks are created; one at 180.8 kHz and a smaller one at 315 kHz (red graph). These changes are likely due to the stiffness loss, decreasing the loading ratio *r*, resulting in a leftward shift of the main peak, as shown in Figure 5a. According to [33], when damage has reached a critical level, new peaks in the spectrum appears, this is consistency with the results observed here.

The signature changes even weeks after the induced damage. From the 3 months after damage signature (yellow graph), it is seen that the peak magnitudes decrease, and is right shifted. This could indicate that the RC sample stiffness is increased after being dried, and the solidification of the rust-products may take place inside the concrete.

Figure 18b, where a patch-size of 10 × 10 × 0.5 mm has been used, shows a small resonant area around 340 kHz before damage (blue graph), after the induced corrosion damage, the main peak moves to 351 kHz (red graph), after 3 month the signature stabilizes at 360 kHz (yellow graph). There are no distinct peaks, as with the 1.5 mm thick patch. This can make identification of changes more difficult.

### 3.5. Reference Reinforced Concrete Sample

**Figure 19:**
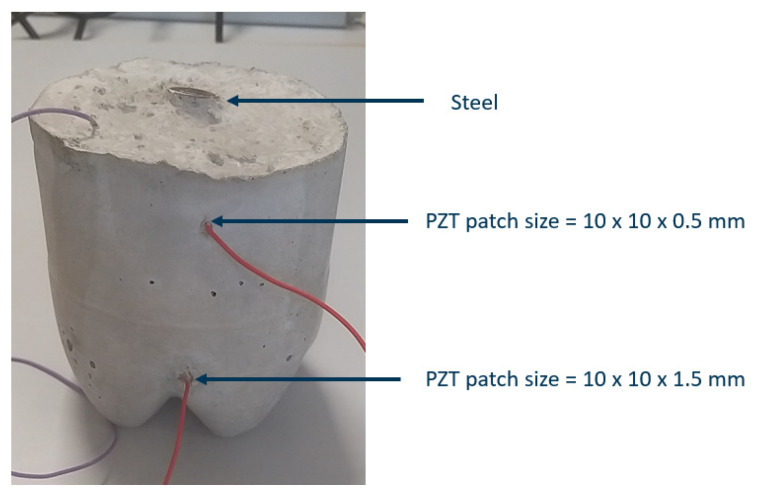
Reinforced concrete with 10 cm long steel rod, used as reference.

**Figure 20:**
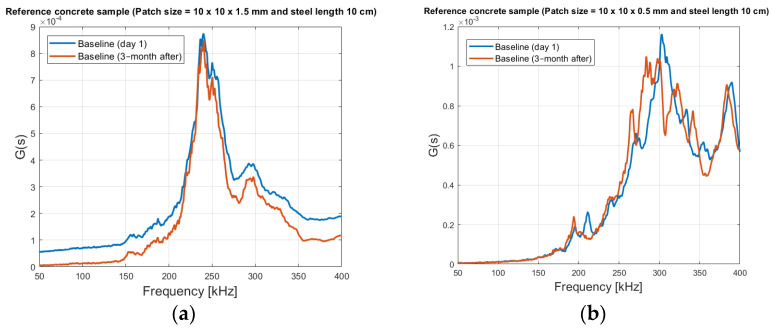
Reference concrete with steel rod with length of 10 cm (**a**) Patch Size of 10 × 10 × 1.5 mm and (**b**) Patch Size of 10 × 10 × 0.5 mm.

As shown in Figure 19, the small 10 cm steel rod embedded in RC was not damaged and was used as a reference point. With the 10 mm × 10 mm × 1.5 mm patch size (placed at the bottom of the concrete), the baseline admittance signature is recorded for day one and again after 3 months as shown in Figure 20a. It shows no significant changes in shape and peak placement, beside a small offset, which can be observed. This shows that some precaution must be taken, when using the whole impedance spectrum as damage quantification, such in the RSMD, because these natural variations in the electrical admittance/impedance, may give false readings. For 10 × 10 × 0.5 mm (placed at the top of the concrete) (see Figure 20b) baseline day 1 and 3-months after signature shows a more unclear picture, where there are some shift-movements and new peak areas, even though no damaged has been introduced to the concrete sample.

## 4. Discussion

The theoretical analysis using 3 models shows how the impedance changes with respect to the loading. Baptista et al.’s model does not take the resonant frequencies into account, but Liang and Giurgiutiu’s et al.’s model does. However, it was found more suitable to mainly use Giurgiutiu’s et al.’s model, due to their good experimental results, and equation-wise that the sensitivity information is packed into one parameter: the loading ratio *r*, as seen in Equation (16).

The model shows what happens to the spectrum including resonant areas, when damage is simulated. Though this is still a rough approximation, the model assumes a square patch, and only takes vibrations in the length direction into account, and not, for example, the vibrations in the thickness direction. The mechanical stiffness/impedance is frequency dependent, which means that even large structures, can have frequency areas with low mechanical stiffness/impedance. In the analysis here the mechanical stiffness/impedance was assumed not to be frequency dependent. For a more detailed analysis COMSOL/ANSYS simulation would be needed, where also the concrete environment should be included.

In the literature a patch size of 0.2 [34], 0.3 mm [19] and 0.5 mm [35] are typically used for corrosion monitoring in RC structures. But the experiments here show that increasing this to 1.5 mm, clear and strong peaks can be obtained, which corresponds with resonant modes in the RC structure. This behavior is desired when looking for changes between the pristine and damaged signature, by using damage metrics index such as the correlation coefficient deviation (CCDM), see Equation (21).

(21) $$CCDM=1-\frac{\sum_{n}^{N}\left(Z_{n,h}-\bar{Z_{h}}\right)\left(Z_{n,d}-\bar{Z_{d}}\right)}{{\sqrt{\sum_{n}^{N}\left(Z_{n,h}-\bar{Z_{h}}\right)}}^{2}{\sqrt{\sum_{n}^{N}\left(Z_{n,d}-\bar{Z_{h}}\right)}}^{2}}$$

where, *Z_n,h_* and, *Z_n,d_* are the electrical (peak) impedance (modulus, real or imaginary), in healthy (pristine) and damaged condition respectively measured at frequency n, where N is total number of frequency components; $\bar{Z_{h}}$ and $\bar{Z_{d}}$ are the average of the healthy and damage impedance value respectively [29].

According to Lim et al. [33] it is sufficient and sometimes more useful to just use the reduction in resonance frequency, as the damage quantifier. Furthermore, Weijie Li. et al. [20] have, through multiphysics simulation and experimentally, shown that there is linearity between the peak frequency (magnitude and shift) and the corrosion amount. The peaks showed a leftward shift with increasing corrosion amounts, due to stiffness and mass loss. However, their experiments were not performed in reinforced concrete or steel rods, but in rectangular metal coupons in free air. Nevertheless, similar observations are seen here, when the 1.5 mm thick patch is used.

Regarding stability, the thicker patches seem to be much more robust and stabile regarding natural variation with time, as seen in Figure 20. This may be due to the lower capacitance. Disadvantages of a thicker patch are, that it is more prone to damages, because there is a larger area that can be damaged.

The level of corrosion damage that can be detected depends on the location of the damage. The farther away the damage is from the patch, the less sensitivity. According to [27], the sensing range in simple metallic beams is 2 m. This will be much lower in reinforced concrete since the wave amplitude would be reduced over such a distance. In the experiments in this paper, in reinforced concrete a volume metal loss of around 800 µm^3^ could be detected 16 cm from the patch location as a leftward shift of around 1 kHz of the impedance peak. However, more investigations are needed regarding this topic.

One important external factor which can influence the impedance signature is the temperature. According to [20,36] higher operation frequencies are more impacted by the temperature. Baptista et al. [36] have shown that a 20 °C temperature increase results in a leftward shift of 1.5 kHz (at 197.80 kHz) of the peak impedance. This can of course make it more difficult to detect smaller damages, especially in reinforced concrete, as we see in this paper, the resonant frequencies are placed above 200 kHz, and thereby would be more sensitive to the temperature impact.

Corrosion issues is mostly significant in marine environment, due to the ingress of chloride ions. Therefore, to simulate this, the RC structure should remain in a salt-water tank for simulating real-world situations involving seawater, and not be dried up, while the measurements are taken, for proper interpretation of the impedance changes. To perform this, cheap portable instrumentation is needed, since many experiments must be performed simultaneously for adequate results. Current impedance analyzers are too expensive and bulky. Therefore, the authors are in the process of developing a novel cheap instrument for EMI measurements.

When comparing the different patch-sizes, reservations must be taken for the variation in the PZT ceramic parameters. According to the manufacturer (American Piezo) up to 20% tolerance can be on some of the parameters of the PZT element [37]. The adhesive layer must also be considered, when comparing the different measurements.

**Figure 21:**
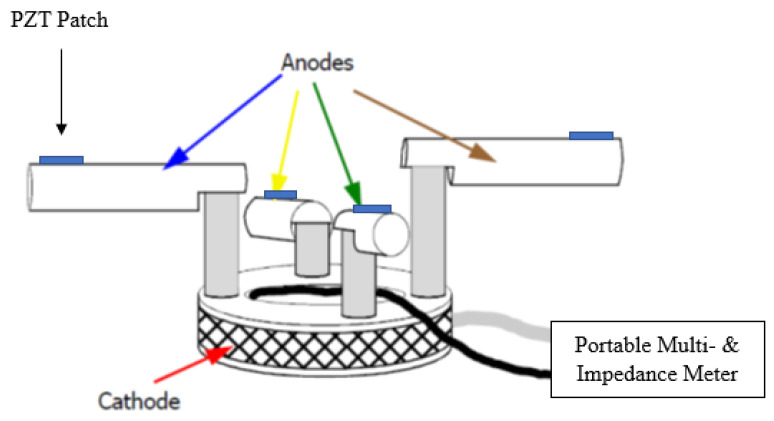
Electromechanical impedance, Voltage and Current measurements using a modified CorroWatch.

In the near future, the authors foresee great opportunities in using EMI technique in conjunction with existing corrosion monitoring systems. In Oresund’s tunnel (SE/DK), the corrosion monitoring system is based on a system called CorroWatch, which is developed by FORCE Technology [38]. It is composed of four anodes (made of steel) and one cathode (made of titanium). When corrosion occurs, the anodes start to corrode, which can be measured through the potential or (macro) current developing between the anode and cathode. By attaching Piezo-electrical patch on a grooved flat surface on the anodes (see Figure 21), electromechanical impedance, voltage and current measurements can be performed for high-level corrosion assessment.

## 5. Conclusions

This paper investigated theoretically and experimentally the optimum patch size thickness for detecting corrosion in reinforced structures. The theoretical analysis matches well with many of the empirical measurements. This indicates that the models can be used for appropriate design of the patch for optimum sensitivity. The results shows that it is important to excite resonant modes to obtain a high level of sensitivity.

With steel rods in free air, all patch size thickness contains peaks. But in reinforced concrete (RC) due to the high pressure (loading) exerted on the patches by the concrete, only the 1.5 mm thick patch were able to generate clear resonant areas. This has to do with the ratio between the mechanical impedance of the host structure and the PZT patch, which if too large, results in no/low resonant areas, and thereby low sensitivity. Increasing the patch thickness increases the sensitivity. Though, the theoretical analysis shows that if the host structure is small, then it is more advantageous to use a smaller patch size.

The investigation also shows that not all resonant modes are automatically sensitive to damages. Therefore, impressed-current acceleration was performed on the RC structure, to induce corrosion damages. It was shown that the 1.5 mm thick patch could indeed detect clear changes in the impedance signature with a leftward-shift and formation of new peaks in the spectrum, indicating significant damage.

The EMI technique is a promising approach that with improvements (e.g., cheaper instrumentation and temperature calibration), can be used as an addition to existing corrosion monitoring systems such as those suggested in the CorroWatch probe.

## Figures and Tables

**Table 1:** PZT patch parameters [18].

Property	Symbol	Value
Compliance, in plane	s_11_	15·10^−12^ Pa^−1^
Dielectric constant	*ε*_33_	1750·*ε*_0_
In-plane induced-strain coefficient	*d*_31_	−175·10^−12^ m/V
Density	*ρ*	7700 kg/m^3^
Mechanical loss factor	*η*	2%
Electrical loss factor	*δ*	1%

Note: *ε*_0_ = 8.85 × 10^−12^ F/m.

## Data Availability

Not applicable.
